# The Overexpression of Insulin-Like Growth Factor-1 and Neurotrophin-3 Promote Functional Recovery and Alleviate Spasticity After Spinal Cord Injury

**DOI:** 10.3389/fnins.2022.863793

**Published:** 2022-04-29

**Authors:** Zuliyaer Talifu, Chuan Qin, Zhang Xin, Yixin Chen, Jiayi Liu, Subarna Dangol, Xiaodong Ma, Han Gong, Zhisheng Pei, Yan Yu, Jianjun Li, Liangjie Du

**Affiliations:** ^1^School of Rehabilitation, Capital Medical University, Beijing, China; ^2^Department of Spinal and Neural Functional Reconstruction, China Rehabilitation Research Center, Beijing, China; ^3^Chinese Institute of Rehabilitation Science, Beijing, China; ^4^Center of Neural Injury and Repair, Beijing Institute for Brain Disorders, Beijing, China; ^5^Beijing Key Laboratory of Neural Injury and Rehabilitation, Beijing, China; ^6^School of Rehabilitation Sciences and Engineering, University of Health and Rehabilitation Sciences, Qingdao, China; ^7^Department of Urology, Beijing Friendship Hospital, Capital Medical University, Beijing, China; ^8^School of Sports Medicine and Rehabilitation, Beijing Sport University, Beijing, China; ^9^Department of Rehabilitation Medicine, Xiangya Hospital, Central South University, Changsha, China

**Keywords:** spinal cord injury, nerve repair, nerve growth factor, NT-3, IGF-1, motor function recovery, spasticity, mechanical pain

## Abstract

**Objective:**

This study was conducted to investigate the effects of the exogenous overexpression of nerve growth factors NT-3 and IGF-1 on the recovery of nerve function after spinal cord injury (SCI) and identify the potential mechanism involved.

**Methods:**

Sixty-four female SD rats were randomly divided into four groups: an SCI group, an adeno-associated viral (AAV)-RFP and AAV-GFP injection group, an AAV-IGF-1 and AAV-NT-3 injection group, and a Sham group. After grouping, the rats were subjected to a 10-week electrophysiological and behavioral evaluation to comprehensively evaluate the effects of the intervention on motor function, spasticity, mechanical pain, and thermal pain. Ten weeks later, samples were taken for immunofluorescence (IF) staining and Western blot (WB) detection, focusing on the expression of KCC2, 5-HT2A, and 5-HT2C receptors in motor neurons and the spinal cord.

**Results:**

Electrophysiological and behavioral data indicated that the AAV-IGF-1 and AAV-NT-3 groups showed better recovery of motor function (*P* < 0.05 from D14 compared with the AAV-RFP + AAV-GFP group; *P* < 0.05 from D42 compared with SCI group) and less spasticity (4–10 weeks, at 5 Hz all *P* < 0.05 compared with SCI group and AAV- RFP + AAV-GFP group) but with a trend for more pain sensitivity. Compared with the SCI group, the von Frey value result of the AAV-IGF-1 and AAV-NT-3 groups showed a lower pain threshold (*P* < 0.05 at 4–8 weeks), and shorter thermal pain threshold (*P* < 0.05 at 8–10 weeks). IF staining further suggested that compared with the SCI group, the overexpression of NT-3 and IGF-1 in the SCI-R + G group led to increased levels of KCC2 (*p* < 0.05), 5-HT2A (*p* < 0.05), and 5-HT2C (*p* < 0.001) in motor neurons. WB results showed that compared with the SCI group, the SCI-R + G group exhibited higher expression levels of CHAT (*p* < 0.01), 5-HT2A (*p* < 0.05), and 5-HT2C (*p* < 0.05) proteins in the L2-L6 lumbar enlargement.

**Conclusion:**

Data analysis showed that the overexpression of NT-3 and IGF-1 may improve motor function after SCI and alleviate spasms in a rat model; however, these animals were more sensitive to mechanical pain and thermal pain. These behavioral changes may be related to increased numbers of KCC2, 5-HT2A, and 5-HT2C receptors in the spinal cord tissue. The results of this study may provide a new theoretical basis for the clinical treatment of SCI.

## Introduction

As a common disabling disease, spinal cord injury can lead to different degrees of impaired motor and sensory functions below the damaged plane. It is currently estimated that 0.78–1.16 million patients are diagnosed with spinal cord injury (SCI) each year globally (James et al., 2019). Spasticity is a common complication of upper motor neuron damage and up to 70% of patients show the symptoms of spasm following SCI (Finnerup, 2017). Furthermore, neuropathic pain is a common complication of SCI; in fact, more than 50% of patients may experience neuropathic pain after an SCI (Burke et al., 2017). Spasticity often leads to chronic pain and deformity of the skeletal muscle system. Furthermore, neuropathic pain can exert dramatic effects on a patient’s mood, sleep, quality of life, cognitive function, recreational activities, and even employment. These factors can exert a dramatic influence on the rehabilitation process (Andresen et al., 2016; Holtz et al., 2017; Tibbett et al., 2020).

Following SCI, the spinal cord undergoes several adaptive morphological and functional changes, including a reduction of serotonin (5-HT) receptors (Murray et al., 2011), the downregulation of ion channels on motor neurons (D’Amico et al., 2014), the downregulation of potassium chloride cotransporter-2 (KCC2; Boulenguez et al., 2010), and the establishment of a continuous inward current in motor neurons (Hultborn et al., 2004). These effects arise below the injury site and are related to a loss of control in the supraspinal structure (including the motor cortex, brainstem reticular structure, red nucleus, and vestibular nucleus) in the spinal cord. Under the combined action of these factors, motor neurons in the anterior horn of the spinal cord develop an excitable state; this is the primary mechanism responsible for limb spasm after spinal cord injury (Bellardita et al., 2017). The appearance of neuropathic pain also includes supraspinal, spinal, and peripheral mechanisms (Shiao and Lee-Kubli, 2018). Some of these pathways may be related to spasticity (Tashiro et al., 2015). Although a variety of molecular mechanisms involved in spasticity and neuropathic pain after spinal cord injury have been identified, a satisfactory treatment plan has yet to be established.

Neurotrophin-3 (NT-3) is an important growth factor with a number of physiological functions, including promoting survival, growth, the differentiation of neurons, and the stimulation of the formation of the axon (Maisonpierre et al., 1990). Studies of the spinal cord circuit have shown that NT-3 reduces the excitability of motor neurons and promotes circuit reorganization in the spinal cord (Uchida et al., 2008; Petruska et al., 2010; Boyce and Mendell, 2014; Duricki et al., 2016). Insulin-like growth factor-1 (IGF-1) is an active substance in the body that is similar to insulin and is a multifunctional cell regulatory factor that plays an essential role in promoting cell differentiation, individual growth, and development (Hodgetts and Harvey, 2017; Allahdadi et al., 2019). In the clinical literature, researchers have found that the levels of IGF-1 are specifically correlated with changes in spasm and skeletal muscle cross-sectional area in patients with SCI (Gorgey and Gater, 2012). However, the role of IGF-I has yet to be verified in animal models.

We hypothesized that IGF-1 is also a regulatory factor that is closely related to spasticity following SCI. Therefore, we hypothesized that NT-3 and IGF-1 can promote the recovery of motor function after SCI and alleviate spasticity and neuropathic pain. We aimed to investigate the potential mechanisms involved at the spinal cord level in a rat spinal cord injury model. We considered it possible that these factors may play a guiding role in the treatment of SCI by nerve growth factor.

## Materials and Methods

### Animals and Experimental Protocol

**FIGURE 1:**
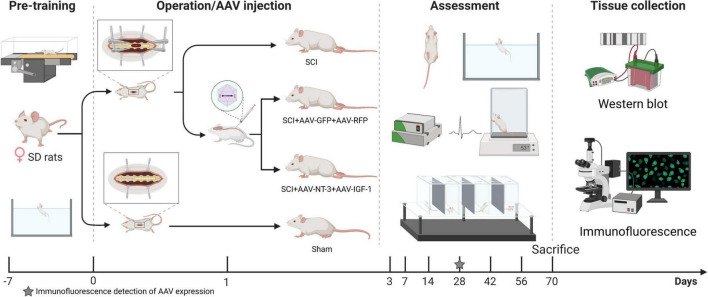
Experimental protocol and time points. After adaptive training for 1 week in advance, the rats were randomly divided into four groups for spinal cord injury modeling or sham operation. Two groups were injected with adeno-associated viral (AAV)-N-T3/AAV-IGF-1 or AAV-GFP/AAV-RFP 1 day after surgery. Three rats were then examined by immunofluorescence 4 weeks after surgery to determine the expression of AAV. The other rats received behavioral and electrophysiological evaluations for 10 weeks. After 70 days, all rats were sacrificed for western blotting and immunofluorescence detection.

A total of 64 female Sprague–Dawley (SD) rats weighing 200–220 g (Beijing HFK Bioscience Co., Ltd., Beijing, China), were included in this experiment. Rats were fed in a standard laboratory animal room and were fed and trained adaptively for 1 week prior to surgery. Rats were randomly divided into four groups: A (SCI group; moderate spinal cord injury at the T9 vertebral level); B [SCI-R + G group; injection of 3 μL adeno-associated viral (AAV)- RFP (1.0 × 10^13^ v.g./ml, HanBio, Shanghai, China) and 3 μL of AAV-GFP (1.0 × 10^13^ v.g./ml, HanBio, Shanghai, China) after SCI as a control group]; C [SCI-N + I group: combined injection of 3 μL AAV-GFP-IGF-1 (5.94 × 10^13^ v.g./ml, HanBio, Shanghai, China) and 3 μL AAV-RFP-NT-3 (6.73 × 10^13^ v.g./ml, HanBio, Shanghai, China) after SCI], and D (Sham group: removal of the lamina of the T9 vertebrae without any other intervention). AAV9 serotype was used in this experiment, which is considered to have a high transfection rate in the nervous system (Brommer et al., 2021). Figure 1 depicts the experimental process. The Ethics Committee approved the experimental protocol and the use of animals in scientific experiments at Capital Medical University (AEEI-2021-149).

### Spinal Cord Injury

The rats were anesthetized with 2% isopentane on a thermal insulation pad (∼37°C). After complete anesthesia, a region (3 cm × 3 cm) of fur was shaved away close to the T9 segment. Then, we used a scalpel to open a process that was 2 cm and ran parallel to the spine with T9 as the center. After complete hemostasis, we separated the muscle and fascia layer and then stopped the bleeding from the paravertebral muscles. We then used bone-biting forceps to remove the spinous process and lamina of the T9 segment and expose an area (3-mm diameter) centered on the spinal cord of the T9 segment and rinsed this area with normal saline. Then, the rats were fixed on a spinal cord impactor (Precision Systems and Instrumentation IH spinal cord impactor, United States); the head and tail of the spinal cord were held in a plane centered on the T9 segment. A striking head for rats was then used to hit the spinal cord with a force of 250 kdyn, thus creating a moderate spinal cord injury model. Following this strike, we observed spinal cord congestion, a spastic left-right swing of the tail, and the retraction of both lower limbs and body, thus indicating that the model had been established successfully. We ensured that the tissue was fully hemostatic and sutured the animals layer by layer with a No. 5 surgical suture needle. Following completion of this procedure, the rats were kept warm until they woke up. Five days after surgery, the rats were given penicillin (12,000 μ/day) to prevent infection. Following surgery, the rats were given manual urinary care every 12 h until spontaneous urination recovered. The rats were euthanized (*via* an excess of pentobarbital injection) if any of the following conditions occurred: the weight loss was >20%, the body temperature was lower than 35°C, or self-mutilation occurred.

### Injection of Adeno-Associated Viral

Twenty-four hours after the SCI operation, we injected AAV, as described previously (Boyce et al., 2012; Asboth et al., 2018). In brief, the rats were fixed on an operating table to expose the rat spinal cord and anesthetized with 2% isopentane. The injection site was located 5 mm from the caudal end of the strike site. A total of 3 μL of AAV virus solution was injected at a speed of 1 μl/min (Quintessential Stereotaxic Injector, United States) close to the midline at a depth of 1 mm (Hamilton 5 μL microsyringe, Bonaduz, Switzerland). The needle was retained *in situ* for 5 min; the injection was deemed successful if no liquid was released when the needle was withdrawn. Following injection, we stopped any bleeding and sutured the site layer by layer. The rats were then placed in a warm environment until they woke up.

### Behavioral Analyses

Basso, Beattie, and Bresnahan (BBB) scales were used to score the hindlimb motor function of rats after SCI: 0 points represented complete paralysis and 21 points represented completely normal function (Diogo et al., 2019). Prior to each BBB evaluation, the rats were placed in the evaluation environment for 15 min in advance to adapt to the environment. The test area was an annular closed area with a diameter of 90 cm that was separated from the outside by a transparent plate with a height of 10 cm. Two independent researchers observed the activities of the rats within 4 min. The hind limb motor function in each rat was evaluated by a digital footprint analysis system (DigiGait™, Mouse Specifics, Inc., Massachusetts, United States). The running speed was slowly increased to 10 cm/s and a high-speed camera was deployed (Basler high speed camera, Ahrensburg, Germany); at least four consecutive gait cycles were evaluated. Three images were recorded for each rat and evaluation.

### Spasticity Analysis

**FIGURE 2:**
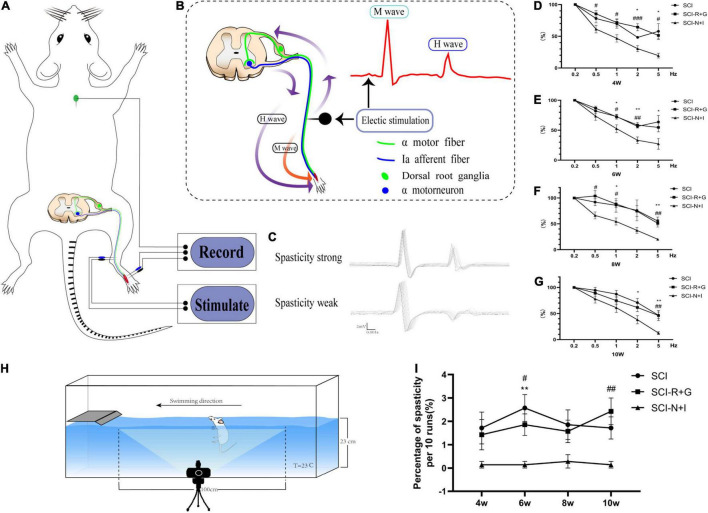
Electrophysiological and behavioral evaluation of spasm. **(A)** A model showing the principle of H-reflex evaluation. The reference electrode was located on the back of the rat’s neck and stimulated the tibial nerve at the upper part of the ankle joint; this allowed us to record electromyography signals from the foot muscle group. **(B)** Schematic diagram of the H-reflex in the spinal cord. When stimulating the tibial nerve, a motor fiber and Ia afferent fiber were excited simultaneously. The electrical signal transmitted by the motor fiber caused muscle contraction; that is, an M wave was recorded by EMG. The Ia afferent fiber reversely excited a motor neuron and then caused muscle contraction through a motor fiber; that is, an H wave was recorded by EMG. **(C)** The most significant difference between normal strong spasmodic rats and strongly spasmodic rats was the much higher H-wave in the latter group. **(D–G)** RDD evaluation results of the electrophysiological H-reflex at 4–10 weeks (*n* = 6, per time point and per group). **(H)** Schematic diagram showing how spasticity was judged during swimming after spinal cord injury. **(I)** The frequency of spasticity/ankle clonus in 10 swimming tests at each time point (*n* = 7, per time point and per group). *, # represents the significance of the difference (SCI-N + I vs. SCI, SCI-N + I vs. SCI-R + G groups at each time point; one-way ANOVA, Bonferroni’s *post hoc* test). One, two, and three symbols indicate *P* < 0.05, *P* < 0.01, and *P* < 0.001, respectively. ns indicates no statistical difference between groups.

We used specific evaluation methods to test the H-reflex RDD. The rats were fixed on the operation table and anesthetized with 2% isopentane. Two 24-g needle electrodes were inserted into the inner side of the lower limb ankle to stimulate the tibial nerve. We then recorded the electrical signals produced at a stimulation wave width of 100 ms (Electronic Stimulator, ADInstruments, New South Wales, Australia). By signal amplification (×4,000) and filtering (passband: 300 Hz to 6 kHz), we used PowerLab software LabChart (Version 8.0, ADInstruments, New South Wales, Australia) to convert the electrical signals and analyze the resultant data. First, we measured the current intensity required to obtain the maximum H wave at a frequency of 0.1 Hz and a wave width of 0.2 ms (the current increased in increments of 0.1 Hz; the measured Hmax at 2–3 mA could then be obtained). Then, to measure the RDD of the H reflex, 20 consecutive stimuli of 0.1, 0.5, 1, 2, and 5 Hz were performed, respectively. The interval between each series of stimuli was 2 min, and each evaluation took about 15 min (as shown in Figure 2). Due to the considerable variation of the first five sets of data in each group, 15 stimuli after each series of incentives were retained to calculate their peak values; we determined the mean value of the last 15 values. For statistical analysis, an average the H wave value at 0.1 Hz was set as the baseline; then, the average *H* value at 0.5, 1, 2, and 5 Hz was divided by the value of 0.1 Hz to calculate the RDD of H reflection (Lee et al., 2014; Kathe et al., 2016; Ryu et al., 2017).

A swimming test was conducted, as previously described (Ryu et al., 2017; Chang et al., 2019). Figure 2H shows how the swimming tests were performed; the apparatus involved a rectangular plexiglass container filled with tap water (150 cm length × 15 cm width × 40 cm height); the depth of the water was 23 cm and the water temperature was maintained at 23°C. During the pre-training, the table was located on the left side of the container; the rats could be controlled to swim continuously from right to left. We recorded the occurrence of spasm in the middle 100 cm of swimming. If there was at least one clonic phase or spasm phase in a single swimming event, then the experiment was recorded as being spasm-positive and the number of positive spasms in 10 evaluations was recorded. The recorded results were abolished if the rats showed defecation or urination-related behaviors during the test. All swimming tests were recorded with a Canon 760D camera.

### Mechanical Pain and Thermal Pain

**FIGURE 3:**
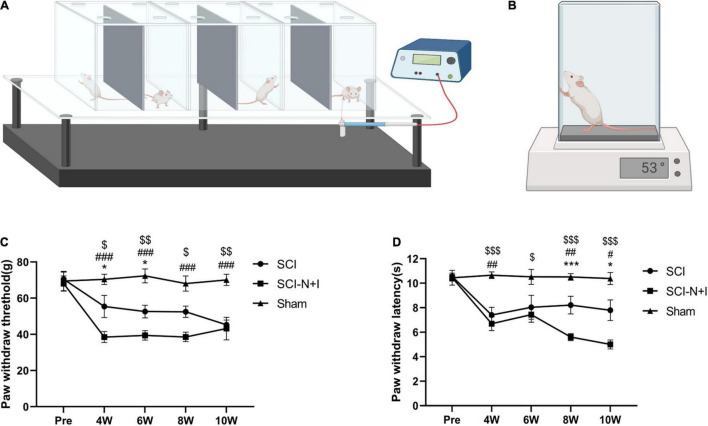
Schematic diagram of neuropathic pain measurements. **(A)** Electronic von Frey evaluation model diagram. Each rat was placed in a transparent cage. Then, we gradually stimulated the soles of the feet through barbed wire at the bottom of the cage. The threshold of foot withdrawal was recorded as an evaluation value. Double measurements were recorded three times and the average value was taken each time. The evaluation interval was 10 min (*n* = 7–10 for the SCI-N + I group and the SCI group, *n* = 6 for the Sham group). Each rat was evaluated three times, and the average value was determined. **(B)** Diagram showing how we performed hot plate assessments on rats using a hot plate preheated to 53 ± 0.1°C. Reaction times (e.g., for jumping) were recorded three times for each rat and the average value was taken at a time interval of 10 min each time (6–8 animals per group were evaluated at each time point; each rat was evaluated three times and the average value was taken). **(C)** The results of von Frey evaluation at baseline and 4–10 weeks after surgery. **(D)** The results of the hot plate evaluation before and 4–10 weeks after surgery. *,#,$ represents the significance of the differences between the SCI vs. SCI-N + I, Sham vs. SCI-N + I, and SCI vs. Sham groups at each time point (one-way ANOVA, Bonferroni’s *post hoc* test). One, two, and three symbols indicate *P* < 0.05, *P* < 0.01, and *P* < 0.001, respectively.

Electronic von Frey methodology (Bioseb, Pinellas Park, FL, United States) was used to evaluate the mechanical pain experienced by the experimental rats, as shown in Figure 3A. The rats were placed in a plastic cage with a metal mesh bottom that could make complete contact with the feet of the rats. Prior to evaluation, the rats were allowed to adapt to the environment for approximately 15 min. During the assessment, a plastic tip connected to a pressure sensor was placed in the middle of the plantar area of the rat’s hind limb, and the application force of the handle was increased slowly and steadily. If the foot retracted or the rat licked or jumped, then a positive reaction was recorded and the force applied when the reaction occurred was registered. Measurements were taken from the left and right feet of each rat on three occasions and the average value was determined. The time interval for each recording was 10 min (Hao et al., 2019). Thermal pain experienced by the rats was evaluated by a hot plate test (Bioseb, Pinellas Park, FL, United States), as shown in Figure 3B. During the evaluation, the rats were placed on a plate heated to 53 ± 0.1°C in advance, and the time taken to escape, such as hindlimb retraction, foot licking, and jumping, was recorded. Each rat was evaluated three times with an interval of 10 min and the average value was determined. To prevent scalding, the maximum test time was 30 s (Yalcin et al., 2009).

### Immunohistochemical Sampling, Fixation, and Section Staining

The rats were anesthetized with sodium pentobarbital (40 mg/kg, i.p.), fixed on an operating table and the skin and sternum were cut successively to expose the heart. First, 0.9% normal saline was used for heart perfusion; then, 4% paraformaldehyde solution was used for fixation. After fixation, the spinal cord tissue of lumbar enlargement (L2 ∼ L6) was removed and stained. The interval between slices was 500 μm, so that each motor neuron appeared in only one section. The tissue sections were quantitatively analyzed by strata Quest (TissueGnostics, version 7.0.1.176), and the cell body diameter of layer IX ChAT-positive neurons was determined using a specific antibody (ab181023, Abcam, Cambridge, United Kingdom; dilution 1:100). Then, the mean fluorescence intensity of KCC2 (ab134300, Abcam, Cambridge, United Kingdom; dilution 1:200) and 5-HT2A (ab216959, Abcam, Cambridge, United Kingdom; dilution 1:100)/5-HT2C (ab133570, Abcam, Cambridge, United Kingdom; dilution 1:100) around the motor neurons was determined. Motoneurons were identified using a red fluorescence (ChAT) channel. Then, we determined the fluorescence intensity of the green fluorescence channel (KCC2/5HT-2AR/5HT2CR) within the recognition range of motoneuron cell bodies. We also determined the co-expression of green fluorescence intensity and motoneurons and the fluorescence intensity of the corresponding marker for each motoneuron was calculated by dividing by the number of motoneurons in a given field (TissueGnostics- StrataQuest 7.1).

### Western Blotting

Western blotting was used to detect the expression levels of NT-3 (ab16640, Abcam, Cambridge, United Kingdom; dilution 1:1000), IGF-1 (ab36532, Abcam, Cambridge, United Kingdom; dilution 1:1000), ChAT (ab181023, Abcam, Cambridge, United Kingdom; dilution 1:1000), 5-HT2A (ab216959, Abcam, Cambridge, United Kingdom; dilution 1:1000), and 5-HT2C (sc-17797, SANT, United States) in the spinal cord of each lumbar enlargement. In brief, the spinal cord tissue from the L5 level (approximately 2.5 mm) was removed under deep anesthesia with sodium pentobarbital. The tissue was then frozen with liquid nitrogen and stored at −80°C for western blotting. Tissue samples were lysed with RIPA buffer (G2002, Servicebio, Wuhan, China) and tissue homogenates were centrifuged at 12,000 rpm for 10 min at 4°C. A BCA protein concentration assay kit (G2026, Servicebio, Wuhan, China) was used to determine protein concentrations. Then, protein samples were denatured in a boiling water bath for 15 min and then stored in a refrigerator at −20°C to await analysis. Next, we conducted SDS-PAGE electrophoresis (G2003, Servicebio, Wuhan, China) and transferred separated proteins to a PVDF (G6015-0.45, Servicebio, Wuhan, China) membrane. Membranes were then probed with primary antibodies overnight at 4°C. The following morning, the membranes were washed three times, incubated with a secondary antibody (GB23303, GB23404, GB23301, GB23302, Servicebio, Wuhan, China, dilution 1:3000), washed, and analyzed by chemiluminescence and gel image analysis.

### Statistical Analysis

All data are given as the mean ± standard error and SPSS software (version 26.0, IBM, Armonk, NY, United States) was used for statistical analysis. Two independent sample *t*-tests were used to compare two groups and one-way ANOVA was used for comparisons between multiple groups, Bonferroni’s *post hoc* test was used for correction. *P* < 0.05 was statistically significant.

## Results

### Adeno-Associated Viral Was Successfully Transfected and Continuously Expressed

**FIGURE 4:**
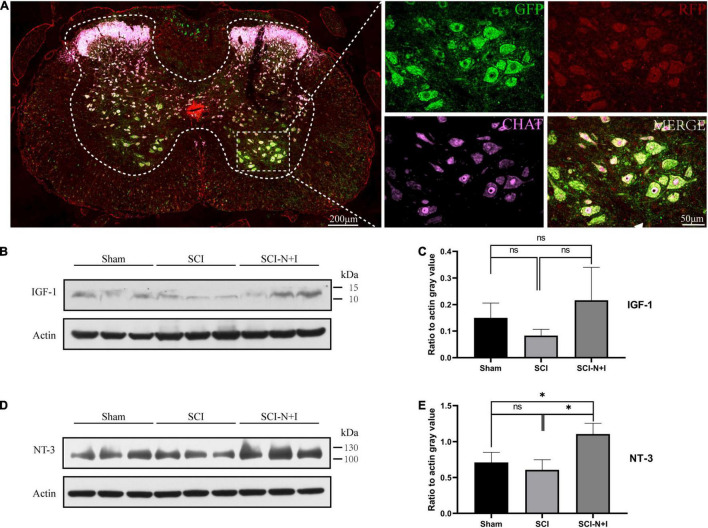
The detection of AAV expression. **(A)** Four weeks after surgery, the spinal cord of the AAV injection group (*n* = 3) was randomly selected for immunofluorescence staining to judge whether AAV had been successfully transfected. **(B,C)** At the end of the experiment, the level of IGF-1 protein in the lumbar distension of the spinal cord was detected by western blotting (*n* = 3 per group). **(D,E)** The level of NT-3 protein in the lumbar distension of the spinal cord was detected by western blotting at the endpoint of the experiment (*n* = 3 per group). *Represents the significance of the difference between groups (one-way ANOVA, Bonferroni’s *post hoc* test). One, two, and three symbols indicate *P* < 0.05, *P* < 0.01, and *P* < 0.001, respectively. ns indicates no statistical difference between groups.

Four weeks after surgery, immunofluorescence analysis detected successful transfection signals in the spinal cord. The red and green fluorescent proteins carried by AAV were strongly co-localized with the motor neuron-specific marker ChAT (Figure 4A). These data indicated that AAV had successfully been transfected into motor neurons in the spinal cord and expressed the target gene. Western blotting after 10 weeks showed that the expression levels of NT-3 and IGF-1 tended to increase in the SCI-N + I group (Figures 4B–E). The statistical results verified that the expression of NT-3 was higher than that in the SCI group (*P* = 0.016) and Sham group (*P* = 0.046). However, due to the significant differences in IGF-1 protein within the group, the protein content of several groups was not statistically significant in the statistical analysis (SCI-N + I vs. SCI-R + G, *p* = 1.000; SCI-N + I vs. SCI, *P* = 0.255; SCI-R + G vs. SCI, *P* = 1.000).

### The Combined Overexpression of Neurotrophin-3 and Insulin-Like Growth Factor-1 Led to the Improved Recovery of Motor Function

**FIGURE 5:**
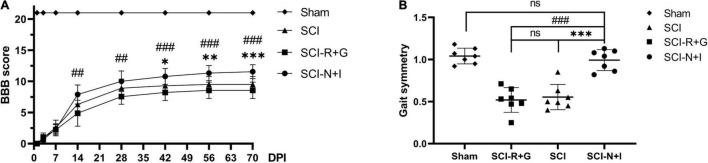
Motor function assessment results. **(A)** The BBB scores of the four groups (*n* = 9 rats per group); one-way ANOVA was conducted between the SCI, SCI-R + G, and SCI-N + I groups. **(B)** The gait symmetry of the four groups was obtained by digital gait analysis in week 10 (*n* = 7 per group). *, # represents the significance of the differences (SCI-N + I vs. SCI, SCI-N + I vs. SCI-R + G at each time point; one-way ANOVA, Bonferroni’s *post hoc* test). One, two, and three symbols indicate *P* < 0.05, *P* < 0.01, and *P* < 0.001, respectively. ns indicates no statistical difference between groups.

Following the combined injection of AAV carrying NT-3 and IGF-1 into the spinal cord of rats with moderate spinal cord injury, we found that the AAV-R + G group showed the worst motor function while the AAV-N + I group showed the best motor function (Figure 5A). There was a significant difference in BBB score (*P* = 0.006) between the AAV-N + I group (7.89 ± 1.54) and the AAV-R + G group (4.89 ± 2.08) after 2 weeks. This difference became more evident from 4 weeks to the endpoint of the experiment. In the sixth week after surgery, there was also a difference in BBB score between the AAV-N + I group and the SCI group (10.78 ± 1.33 vs. 9.30 ± 1.33) (*P* = 0.014). At the end of the experiment, the BBB score of the SCI-N + I group (11.56 ± 1.13) was higher than that of the SCI group (9.50 ± 1.27) and SCI-R + G group (8.56 ± 1.333) (SCI-N + I vs. SCI-R + G, *p* < 0.001; SCI-N + I vs. SCI, *P* < 0.001; SCI-R + G vs. SCI, *P* = 0.698). Digit gait evaluation found that 10 weeks after surgery, the gait symmetry of the SCI-N + I group (0.99 ± 0.047) had significantly improved when compared with the SCI group (0.55 ± 0.057; *P* < 0.001) and the SCI-R + G group (0.52 ± 0.056; *P* < 0.001); there was no significant difference between the SCI-N + I group and the Sham group (1.04 ± 0.035; *P* = 1.00) (Figure 5B). Collectively, the BBB score and digit gait analysis suggested that AAV treatment led to a great improvement in motor function.

### The Effect of Neurotrophin-3 and Insulin-Like Growth Factor-1 Overexpression on Lower Limb Spasm

From 4 to 10 weeks after surgery, the SCI-R + G group and the SCI group had a higher RDD of the H wave than the AAV-N + I group. This trend was more noticeable at higher frequencies; in the SCI-N + I group, the proportion of H waves at 5 Hz frequency was around 20% while that for the SCI group and SCI-R + G group were around 50% (at 5 Hz, all *P* < 0.05, except for SCI-N + I vs. SCI-R + G at 6 weeks, *P* = 0.153) (Figures 2D–G). Furthermore, swimming tests showed that the SCI-N + I group had a lower frequency of spasm (0.14 ± 0.14% at 4, 6, and 10 weeks) (0.29% ± 0.29 at 8 weeks) while that for the SCI group and SCI-R + G group all exceeded 1.4% (SCI-N + I vs. SCI, *P* = 0.174, 0.003, 0.106, 0.061; SCI-N + I vs. SCI-R + G, *P* = 0.345, 0.035, 0.106, 0.0049 at 4, 6, 8, and 10 weeks) (Figure 2I).

### The Effect of Combined Neurotrophin-3 and Insulin-Like Growth Factor-1 Overexpression on Mechanical Pain and Thermal Pain

As shown in Figure 3C, the von Frey measurement results before surgery in the Sham group were around 70 g; the foot contraction time in response to thermal pain was around 10 s. After spinal cord injury, the mechanical pain and thermal pain threshold both decreased (Sham vs. SCI-N + I, Sham vs. SCI; all *P* < 0.05 after surgery). Analysis showed that 4–6 weeks after the operation the pain threshold of the SCI-N + I group was lower than that of the SCI group (*P* = 0.024, 0.046); this significance gradually disappeared by 8 weeks and 10 weeks (*P* = 0.0504, 1). As shown in Figure 3D, the foot contraction time in response to thermal pain before surgery in the Sham group was around 10 s. After spinal cord injury, the mechanical and thermal pain threshold decreased (Sham vs. SCI-N + I, Sham vs. SCI; all *P* < 0.05 after surgery, except *P* = 0.12 at 6 weeks for Sham SCI). At 4–6 weeks, the hot plate result showed no significant difference between the SCI-N + I and SCI groups (*P* = 1.00). However, there were differences in the evaluation results from 8 to 10 weeks, indicating that the pain threshold in the SCI-N + I group was lower (*P* < 0.001 at 8 weeks and *p* = 0.015 at 10 weeks).

### The Effect of Combined Neurotrophin-3 and Insulin-Like Growth Factor-1 Overexpression on Potassium Chloride Cotransporter-2 Expression in the Membrane of Motor Neurons

**FIGURE 6:**
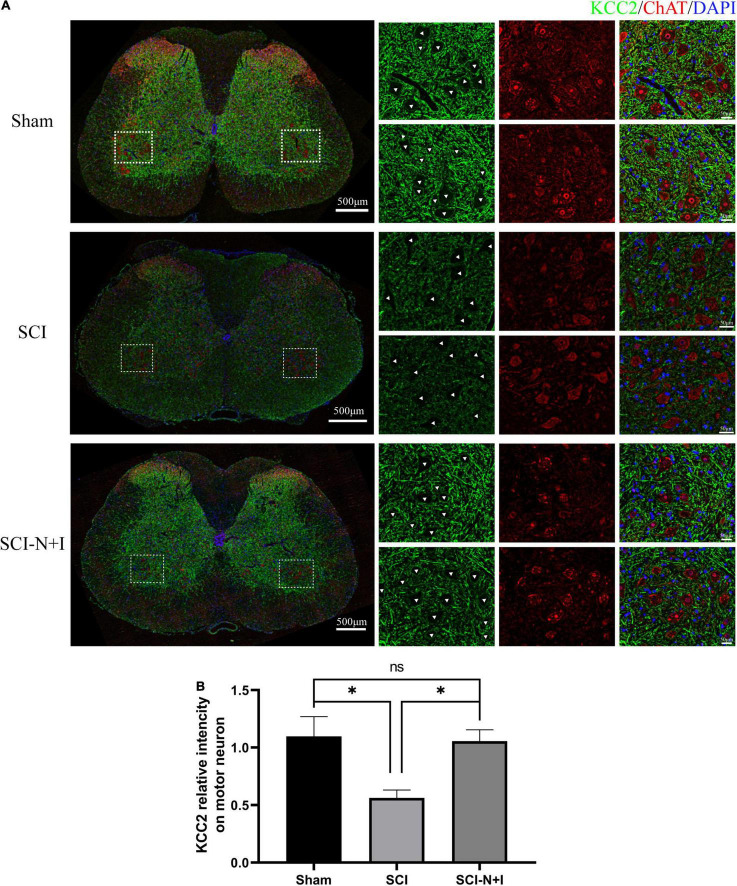
Expression of KCC2 in spinal motoneurons. **(A)** Immunofluorescence staining of KCC2 and ChAT in the spinal cord of rats from different groups. **(B)** Co-localization immunofluorescence intensity of KCC2 and motoneurons, six rats per group (309 ChAT-positive neurons in 22 visual fields in the sham group, 372 ChAT-positive neurons in 23 visual fields in the SCI group, 404 ChAT-positive neurons in 23 visual fields in the SCI-N + I group). *Represents the significance of the difference between groups (one-way ANOVA, Bonferroni’s *post hoc* test), One, two, and three symbols indicate *P* < 0.05, *P* < 0.01, and *P* < 0.001, respectively.

**FIGURE 7:**
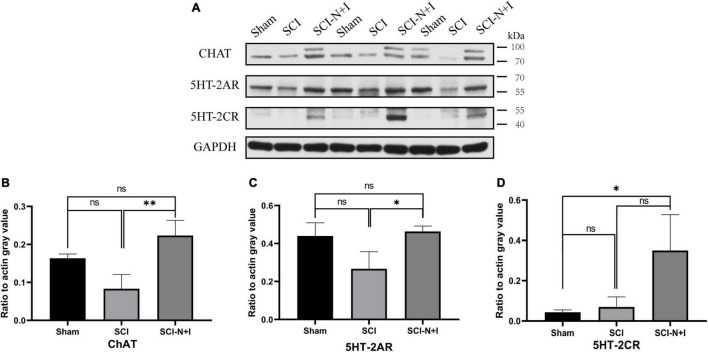
Protein expression of lumbar enlargement at 70 dpi. **(A)** Western blotting of the spinal lumbar enlargements in different groups of rats (*n* = 3 per group). **(B)** Motoneuron marker ChAT, **(C)** 5-HT2A, **(D)** 5-HT2C. *Represents the significance of the difference between groups (one-way ANOVA, Bonferroni’s *post hoc* test). One, two, and three symbols indicate *P* < 0.05, *P* < 0.01, and *P* < 0.001, respectively. ns indicates no statistical difference between groups.

The fluorescence staining of ChAT and KCC2, specific motor neuron markers of the lumbar spinal cord, showed that more motor neurons survived in the NT-3 and IGF-1 treatment group than in the SCI group, as shown in Figures 6A,B. KCC2 fluorescence labeling suggested that the overexpression of NT-3 and IGF-1 increased the levels of KCC2 on the motor neuron membrane and the expression of KCC2 in the cell membrane appeared to be more uniform (SCI-N + I vs. SCI, Sham vs. SCI, *P* = 0.014, 0.007, respectively). As illustrated in Figures 7A,B, western blotting detection also confirmed that the expression of ChAT protein in the AAV-N + I group was significantly higher than that in the SCI group (*P* = 0.006), and reached a level similar to that of the sham group (*P* = 0.196), there were no significant differences between the SCI group and Sham group/Sham group and SCI-N + I group (*P* = 0.072).

### The Effects of Combined Neurotrophin-3 and Insulin-Like Growth Factor-1 Overexpression on 5-HT2A Receptors in Motor Neurons

**FIGURE 8:**
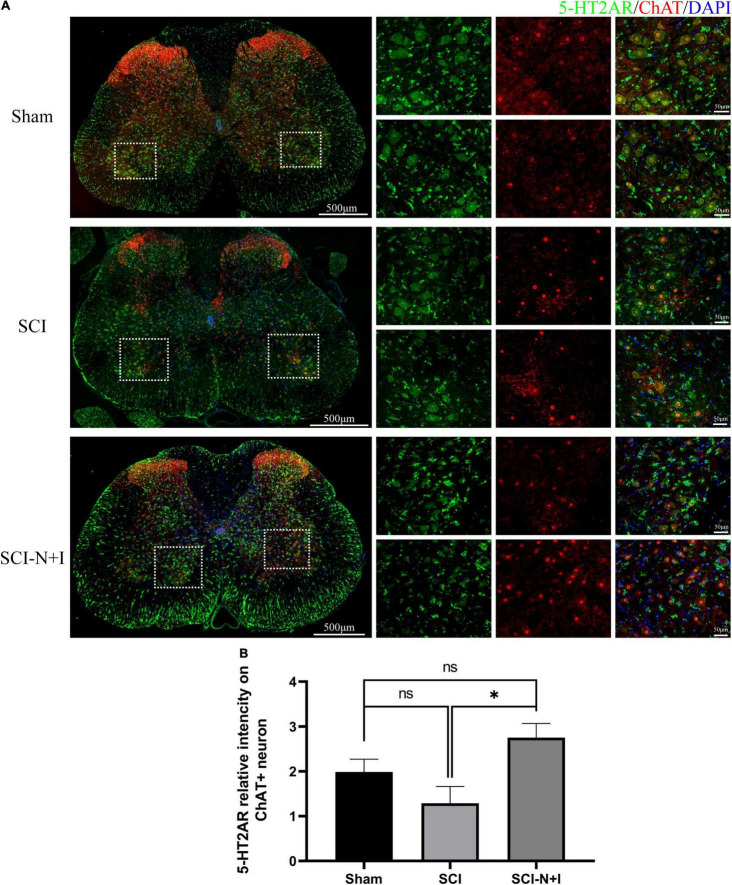
Expression of 5-HT2AR in spinal motoneurons. **(A)** Immunofluorescence staining of 5HT-2CA and ChAT in the spinal cord of rats from different groups. **(B)** Co-localization density of 5-HT2AR and motoneurons, *n* = 6 SCI-N + I group, *n* = 3 in SCI group, and *n* = 4 in the sham group (130 ChAT-positive neurons in eight visual fields in the sham group, 96 ChAT-positive neurons in six visual fields in the SCI group, 292 ChAT-positive neurons in 17 visual fields in the SCI-N + I group). *Represents the significance of the difference between groups (one-way ANOVA, Bonferroni’s *post hoc* test). One, two, and three symbols indicate *P* < 0.05, *P* < 0.01, and *P* < 0.001, respectively.

As shown in Figures 8, 7A,C, immunofluorescence staining and western blotting showed that after spinal cord injury, the expression of 5-HT2A was downregulated in the SCI, SCI-N + I group. The overexpression of NT-3 and IGF-1 significantly increased the levels of 5-HT2C in the lumbar spinal enlargement. The expression levels of the 5-HT2A receptor were higher than in the sham group (SCI-N + I vs. Sham, SCI-N + I vs. SCI, *P* = 0.036, 0.061, respectively). A similar trend was found by co-staining the 5-HT2A receptor and motor neuron marker CHAT. The overexpression of NT-3 and IGF-1 increased the expression levels of the 5-HT2C receptor in motor neurons (SCI-N + I vs. SCI, *P* = 0.033). There was no significant difference in the expression levels of the 5-HT2C receptor when compared between the SCI group and the Sham group (*P* = 0.011).

### The Effects of Combined Neurotrophin-3 and Insulin-Like Growth Factor-1 Overexpression on 5-HT2C Receptors in Motor Neurons

**FIGURE 9:**
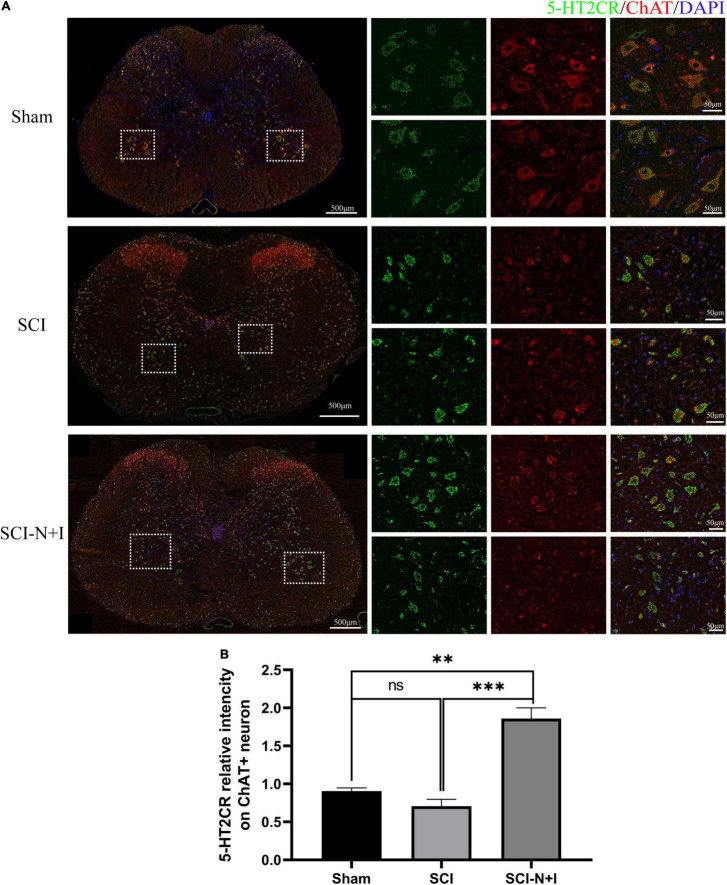
The expression of 5-HT2CR in spinal motoneurons. **(A)** Immunofluorescence staining of 5H-2CR and ChAT in the spinal cords of rats from different groups. **(B)** Co-localization density of 5-HT2CR and motoneurons, *n* = 6 in the SCI and SCI-N + I groups, respectively, *n* = 3 in the sham group (108 ChAT-positive neurons in six visual fields in the sham group, 803 ChAT-positive neurons in 13 visual fields in the SCI group, 787 ChAT-positive neurons in 18 visual fields in the SCI-N + I group). *Represents the significance of the difference between groups (one-way ANOVA, Bonferroni’s *post hoc* test). One, two, and three symbols indicate *P* < 0.05, *P* < 0.01, and *P* < 0.001, respectively.

As shown in Figures 9, 7A,D, immunofluorescence staining and western blotting detection, we showed that under normal conditions and after SCI, the expression levels of 5-HT2C were relatively low; the overexpression of NT-3 and IGF-1 significantly increased the levels of 5-HT2C in the spinal cord. The expression levels of the 5-HT2C receptor in the lumbar enlargement (SCI-N + I vs. Sham, SCI-N + I vs. SCI, *P* = 0.038, 0.055, respectively). Co-staining of the 5-HT2C receptor and the motor neuron marker CHAT also showed a similar trend. The overexpression of NT-3 and IGF-1 significantly increased the expression levels of the 5-HT2C receptor in motor neurons. However, in the SCI group, there was no significant difference in expression levels when compared to the Sham group (SCI-N + I vs. Sham, SCI-N + I vs. SCI, *P* < 0.001, =0.012, respectively).

## Discussion

In this study, we injected an AA vector carrying NT-3 and IGF-1 into the spinal cord of rats after SCI and found that this method promoted the recovery of motor function and reduced the occurrence of spasm. Furthermore, animals were more sensitive to mechanical pain and thermal pain. Injection of the vector also significantly increased the expression of KCC2 in the motor neuron membrane and levels of the 5-HT2A/2C receptor. This may be related to the neuroprotective effect of nerve growth factors, the reorganization of neural circuits in the spinal cord, and the upregulation of the expression of related channel proteins.

### The Role of Neurotrophin-3 and Insulin-Like Growth Factor-1 in Spinal Circuits

Over recent years, the role of various growth factors has been evaluated in spinal cord injury, particularly in animal models. The neurotrophic factor is known to be highly expressed in the developing brain and spinal cord. When this factor matures, its expression level will decrease. However, following SCI, the microenvironment within the spinal cord undergoes dramatic changes. The proportion of neurotrophic factors and their precursors will undergo notable changes and the expression levels of neurotrophic factors will also change as the pathological condition progresses (Fan et al., 2018).

Using models of SCI, it has been established that the high expression levels of fibroblast growth factor (FGF), epidermal growth factor (EGF), glial cell-derived neurotrophic factor (GDNF), and other nerve growth factors are essential for the regeneration of damaged spinous processes (Anderson et al., 2018; Zhu et al., 2020). The local injection of high concentrations of different combinations of nutritional factors [e.g., brain-derived neurotrophic factor (BDNF), IGF-1, GDNF, nerve growth factor (NGF), and NT-3] into the spinal cord during the acute stage of SCI has been shown to reduce the damage incurred by the blood spinal cord barrier, reduce edema and cell injury, and result in an improved functional prognosis (Sharma, 2007). Following injury, motor function training can increase the expression levels of endogenous NGF, NT-3, and IGF-1 in the spinal cord, inhibit cell apoptosis and result in a better recovery of motor function (Jung et al., 2014).

Neurotrophin-3 is known to participate in various physiological processes, such as promoting the survival, growth, and differentiation of neurons and stimulating the formation of axons (Maisonpierre et al., 1990). In the spinal cord circuit, NT-3 can reduce the excitability of motor neurons and promote the regeneration of myelinated axons, effectively promoting the circuit reorganization of the spinal cord (Bloch et al., 2001; Petruska et al., 2010; Boyce and Mendell, 2014). At a higher level, the expression of NT-3 can promote the repair of the corticospinal tract in an animal model of corticospinal tract damage and the recovery of motor and sensory function in an animal model of stroke (Duricki et al., 2016, 2019). More importantly, the relative levels of NT-3 and its receptor trkC are critical for dendritic formation (Joo et al., 2014). Promoting the expression of NT-3 in the spinal cord can significantly increase the number of motor neurons in the anterior horn of the spinal cord and the germination and prolongation of axons (Uchida et al., 2008). Promoting the expression of NT-3 in muscle can improve the prognosis of SCI by regulating specific signal inputs and balancing excitatory and inhibitory neural networks (Kathe et al., 2016). In addition, the nerve repair effect associated with the sustained release of NT-3 through the chitosan carrier has been verified *in vitro* and in the nervous systems of rodents and primates (Li et al., 2009; Yang et al., 2015; Rao et al., 2018).

Insulin-like growth factor-1 is an active substance in the body that is similar to insulin. IGF-1 is a multifunctional cell regulatory factor that plays an essential role in promoting cell differentiation and incrementing individual growth and development (Gibney et al., 2007). IGF-1 is an important survival factor after SCI and can effectively inhibit the spontaneous apoptosis of nerves after SCI, induce the robust regeneration of corticospinal tract axons, and also lead to improved functional recovery (Liu et al., 2017). In an animal model of amyotrophic lateral sclerosis (ALS), it was previously found that regulating the level of IGF-1 could reduce motor neuron death, delay the course of the disease, and delay the occurrence of muscle atrophy (Lin et al., 2018). A previous clinical study also reported that IGF levels might be associated with spasticity after SCI (Gorgey and Gater, 2012).

Our present research shows that the overexpression of NT-3 and IGF in the spinal cord can lead to the improved recovery of motor function and reduce the degree of spasm. The evaluation of mechanical pain and thermal pain revealed a trend for greater sensitivity in the treatment group, although the evaluation trends for mechanical pain and thermal pain were not consistent. We think this may be related to the repair of different pathways after spinal cord injury (since the neural signals of mechanical pain and thermal pain are conducted by different pathways). More importantly, the AAV-NT-3 + IGF-1 group had better motor function recovery, while the mechanical pain and thermal pain evaluation were mainly observed at the time of hindfoot withdrawal. We, therefore, think it may also be a better motor function that led to the shorter paw withdrawal time in this group. In this study, we focused more on motor neurons (mainly concentrated in the forefoot region of the spinal cord); the occurrence of neuralgia may be related to sensory neurons (mainly concentrated in the dorsal horn). We also need to consider that there is a large difference in the way that pain is evaluated in animal experiments and clinical practice. We speculate that these changes may be directly related to the changes in neural plasticity brought about by nerve growth factors.

### The Role of Potassium Chloride Cotransporter-2 After Spinal Cord Injury

In recent years, the role of KCC2 in the central nervous system has received increasing levels of attention. As a neuron-specific transporter, KCC2 is a crucial component of neuronal homeostasis (Bilchak et al., 2021). The dynamic regulation of the KCC2, NKCC1, and GABA systems can maintain the excitability of cells within the normal range by regulating the concentration of chloride ions within cells (Tillman and Zhang, 2019). Following SCI, the level of KCC2 below the injured segment decreases; the downregulation of KCC2 in the motor neuron membrane depolarizes the balance in chloride ion (CI^–^) potential and reduces the intensity of postsynaptic inhibition (Kaila et al., 2014). Recent studies have shown that the overexpression of KCC2 in inhibitory interneurons can partially restore the motor function of mice with complete SCI by forming relay connections (Chen et al., 2018). Following SCI, the overexcitation of the spinal cord reflex, and the reduction in inhibitory synaptic signals, are directly related to the occurrence of spasm (Boulenguez et al., 2010). The hyperexcitability of the spinal cord after SCI is closely related to the damage in descending 5-HT fibers and the transformation of GABAergic interneurons. The downregulation of KCC2 after SCI may represent the common link between these two regulatory imbalances (Wenner, 2014; Sanchez-Brualla et al., 2018). BDNF, as an upstream regulatory factor, can improve nociceptive sensitization by regulating KCC2 expression and GABA excitability (Huang et al., 2017; Romeo-Guitart and Casas, 2020; Ma et al., 2021). Our study also observed that the overexpression of IGF and NT-3 led to better motor function (reached the level of partial weight-bearing walking), and alleviation of spasticity. In our studies, we labeled KCC2 on the membranes of motor neurons in the lumbar enlargement; this molecule is responsible for lower limb movement and was also upregulated. Therefore, we speculate that the improvement of motor function and the alleviation of spasm may be related to the upregulation of KCC2 on motor neurons.

### The Effects of 5-HT Axons and Receptors on Spinal Circuit

Normal spinal cord motoneurons will undergo corresponding continuous discharges in response to synaptic signal inputs; in particular, the constant inward calcium and sodium currents are activated by low voltages (Hultborn et al., 2004). However, the damaged segment can only discharge briefly after SCI. With the slow recovery of function, the continuous inward current will recover slowly. This recovery is significant for the recovery of motor function because it may restore the constant discharge of motor neurons (Nielsen et al., 2007). On the other hand, this recovery may also lead to excessive activity due to the weakening of descending inhibition of the supraspinal structure. The 5-HT axons originating from the brain stem may also play an indispensable role in regulating spinal cord excitability by regulating a variety of receptors (Murray et al., 2011). The spontaneous recovery of motor function after SCI may be related to the remodeling of the cortical reticular spinal cord circuit along with the sprouting and recovery of 5-HT axons (Asboth et al., 2018). Previous research found that the activation of the 5-HT2A subtype receptor could promote the expression of KCC2 on the cell membrane and restore the endogenous inhibition of motoneurons (Bos et al., 2013; Sanchez-Brualla et al., 2018) and the recovery of motor function. The occurrence of spasm may be related to changes in the structure and activity of 5-HT2B and 5-HT2C structures, which slowly restore the recovery of sustained inward current on motor neurons (Murray et al., 2011). Similarly, our research also found that the overexpression of IGF and NT-3 significantly increased the expression of 5-HT2A and 5-HT2C receptors in motor neurons. It is worth noting that the expression levels of the 5-HT2A receptor before injury was relatively high while the levels of the 5-HT2C receptor were lower. The increase in the expression of the 5-HT2C receptor after treatment is highly significant, thus suggesting that this factor may have greater potential in terms of injury and treatment.

### Limitations of This Study

In this study, AAV was *in situ* injected into the spinal cord to achieve the purpose of overexpressing the two target genes, but according to our observations, the motor function of the group injected AAV with fluorescent protein v had a worse trend. We speculate that this is related to the mechanical damage to the spinal cord caused by the *in situ* injection itself. Therefore, more attention should be paid to the choice of administration methods in subsequent studies. Due to the complexity of the central nervous system, the recovery of nerve function after SCI is slow and complex. Consequently, it is difficult to determine which pathway is the most important repair mechanism. Therefore, we cannot exclude other potential pathways/proteins that may contribute to the recovery of motor function, the reduction of spasticity, and the emergence of increased mechanical allodynia and thermal pain sensitivity. In particular, the role of the neurons in the spinal dorsal horn deserves more in-depth exploration. Figure 4A shows that in addition to the motor neurons of the forefoot (ChAT+ neurons with a diameter greater than 40 μm), the red fluorescence is also highly expressed in the hindfoot area. In subsequent experiments, the transfected cell type in the dorsal horn area and its role in the pathological process after SCI deserve to be explored.

Furthermore, the spinal cord is still in the stage of neurological recovery after 10 weeks of injury. Therefore, further investigation is needed to identify other possible mechanisms and provide more information to assist the clinical treatment of SCI. *In vivo* transfection of AAV, the detection time point may be a critical factor, as the AAV transfection efficiency may change over time (Brommer et al., 2021). In this study, we only detected the expression levels of the proteins regulated by the two target genes at 10 weeks. As a result, the NT-3 level was still high (Figures 4D,E), while there was no statistically significant difference in the expression level of IGF-1 (Figures 4B,C). We speculate that this may be mainly due to the low expression level of IGF-1 in the spinal cord, the peak of AAV expression has passed, and the number of animals is small. Nevertheless, combined with the immunofluorescence result at 4 weeks (Figure 4A), it can be confirmed that the AAV transfection was successful and did not affect the conclusion of this study.

In this study, in addition to electrophysiology, the swimming test was also used to assess the spasm. However, we found that the rate of spasticity in swimming in rats in our evaluation does not seem to be as high as in the literature (Ryu et al., 2017; Chang et al., 2019). Based on our experience, this may be mainly due to the differences in rat strains, spinal cord methods (injured segment, surgical method), and evaluation environment (the water temperature remains the same in different experiments, but there may be some differences in room temperature). In addition to this, spasticity mainly involves adaptive changes in the forefoot area of the spinal cord, while neuralgia is more associated with changes in the dorsal horn area. To explain changes in pain perception, more research should be carried out in the sensory input areas of the spinal cord (Shiao and Lee-Kubli, 2018). At present, pain measurement mostly depends on induced pain, such as the von Frey and hot plate tests used in this study. An involuntary paw withdrawal response to pain is also present in animal models of complete SCI (M’Dahoma et al., 2014); this evoked pain may be more applicable to the assessment of spastic syndrome states than pain states (Baastrup et al., 2010). This difference may stem from the more developed spinal autonomic nervous system in rodents and the influence of weight change after surgery (Van Gorp et al., 2014). Moreover, factors, such as weight change after operation, may affect the relevant evaluation results (Turner et al., 2019). According to clinical research, the pain experienced by patients with SCI is more related to chronic pain caused by continuous subthreshold pain. Appropriate evaluation methods need to be investigated further (Wrigley et al., 2018).

## Conclusion

Our research showed that the overexpression of NT-3 and IGF-1 promoted the recovery of motor function and alleviated spasms after SCI in rats; in contrast, there was an increase in sensitivity to pain perception. These trends were accompanied by increased levels of KCC2 and 5-HT2A/5-HT-2C in motor neurons.

## Data Availability Statement

The original contributions presented in the study are included in the article/Supplementary Material, further inquiries can be directed to the corresponding author.

## Ethics Statement

The animal study was reviewed and approved by the Ethics Committee of Capital Medical University.

## Author Contributions

LD and JJL conceived and supervised the study. LD, JJL, YY, ZP, and ZT designed the experiments and provided technical support. ZT, CQ, ZX, YC, JYL, SD, XM, and HG performed the experiments. ZT, CQ, and ZX analyzed the data. ZT wrote the manuscript. LD, ZX, and CQ revised the manuscript. All authors read and approved the manuscript.

## Conflict of Interest

The authors declare that the research was conducted in the absence of any commercial or financial relationships that could be construed as a potential conflict of interest. The reviewer, YW, declared a shared parent affiliation, with the authors, ZT, CQ, YC, JYL, SD, XM, HG, YY, JJL, and LD, to the handling editor at the time of the review.

## Publisher’s Note

All claims expressed in this article are solely those of the authors and do not necessarily represent those of their affiliated organizations, or those of the publisher, the editors and the reviewers. Any product that may be evaluated in this article, or claim that may be made by its manufacturer, is not guaranteed or endorsed by the publisher.

## Supplementary Material

Supplementary Video 1 Spasm/clonus during the swimming test.
