# Supplementary material for: The Overexpression of Insulin-Like Growth Factor-1 and Neurotrophin-3 Promote Functional Recovery and Alleviate Spasticity After Spinal Cord Injury
Source: Front Neurosci. 2022 Apr 29;16:863793. doi: 10.3389/fnins.2022.863793 (PMC9099063; doi:10.3389/fnins.2022.863793)
Supplement: Supplementary file 2 [file Image_1.pdf]

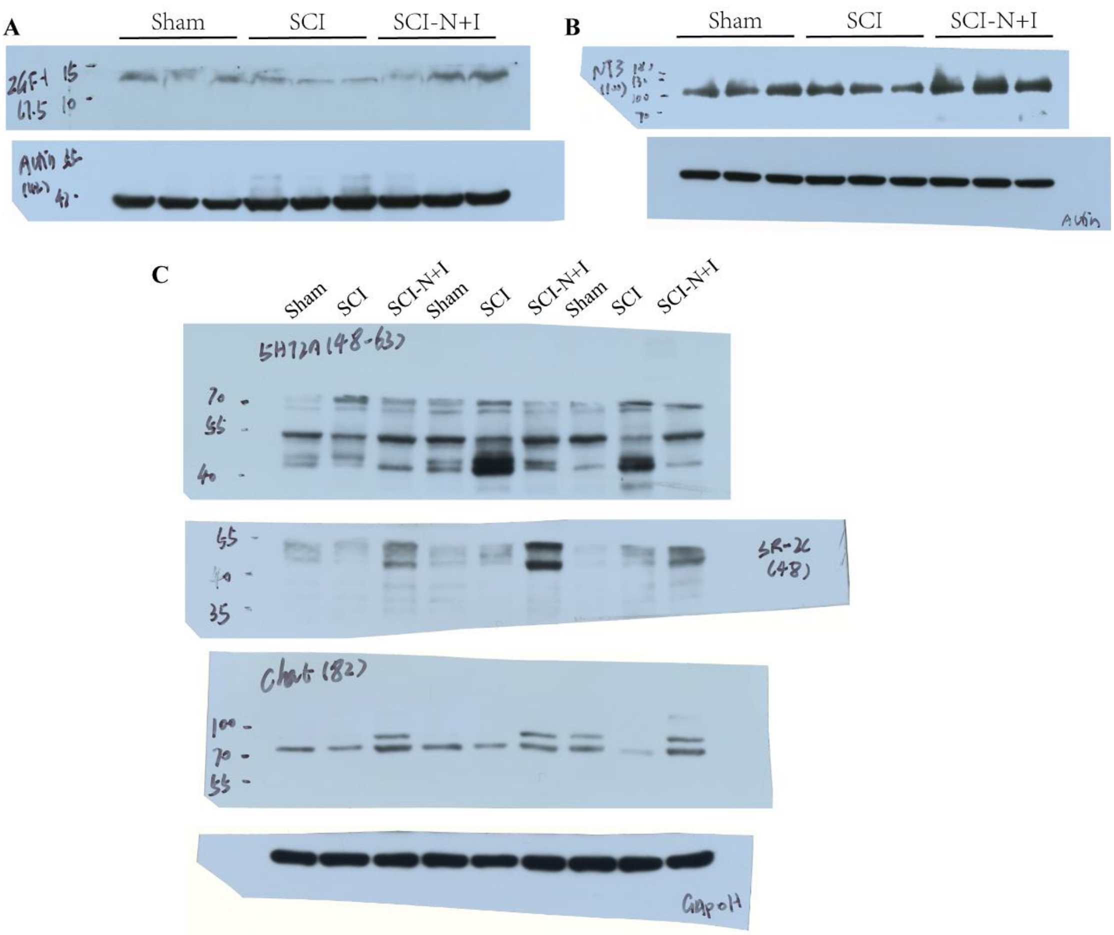

Supplementary Figure 1. Western blots used for analysis in the article. **(A)** Whole images of Western blot membranes as shown in Fig. 2C. **(B)** Whole images of Western blot membranes as shown in Fig. 2E. **(C)** Whole images of Western blot membranes as shown in Fig. 6E, Fig.7E and Fig.8E.
